# MR and CT imaging characteristics and ablation zone volumetry of locally advanced pancreatic cancer treated with irreversible electroporation

**DOI:** 10.1007/s00330-016-4581-2

**Published:** 2016-09-22

**Authors:** Laurien G. P. H. Vroomen, Hester J. Scheffer, Marleen C. A. M. Melenhorst, Marcus C. de Jong, Janneke E. van den Bergh, Cornelis van Kuijk, Foke van Delft, Geert Kazemier, Martijn R. Meijerink

**Affiliations:** 1 Department of Radiology and Nuclear Medicine, VU University Medical Center, Boelelaan 1117, 1081 HV Amsterdam, The Netherlands; 2 Department of Gastroenterology and Hepatology, VU University Medical Center, Boelelaan 1117, 1081 HV Amsterdam, The Netherlands; 3 Department of Surgery, VU University Medical Center, Boelelaan 1117, 1081 HV Amsterdam, The Netherlands

**Keywords:** Pancreatic neoplasms, Ablation, Magnetic resonance imaging, Computed tomography, Tumour volume

## Abstract

**Objectives:**

To assess specific imaging characteristics after irreversible electroporation (IRE) for locally advanced pancreatic carcinoma (LAPC) with contrast-enhanced (ce)MRI and ceCT, and to explore the correlation of these characteristics with the development of recurrence.

**Methods:**

Qualitative and quantitative analyses of imaging data were performed on 25 patients treated with percutaneous IRE for LAPC. Imaging characteristics of the ablation zone on ceCT and ceMRI were assessed over a 6-month follow-up period. Contrast ratio scores between pre- and post-treatment were compared. To detect early imaging markers for treatment failure, attenuation characteristics at 6 weeks were linked to the area of recurrence within 6 months.

**Results:**

Post-IRE, diffusion-weighted imaging (DWI)-b800 signal intensities decreased in all cases (*p* < 0.05). Both ceMRI and ceCT revealed absent or decreased contrast enhancement, with a hyperintense rim on ceMRI. Ablation zone volume increase was noted on both modalities in the first 6 weeks, followed by a decrease (*p* < 0.05). In the patients developing tumour recurrence (5/25), a focal DWI-b800 hyperintense spot at 6 weeks predated unequivocal recurrence on CT.

**Conclusion:**

The most remarkable signal alterations after pancreatic IRE were shown by DWI-b800 and ceMRI. These early imaging characteristics may be useful to establish technical success and predict treatment outcome.

***Key Points*:**

• *This study describes imaging characteristics after irreversible electroporation (IRE) for pancreatic adenocarcinoma.*

• *Familiarity with typical post-IRE imaging characteristics helps to interpret ablation zones.*

• *Post-IRE, no central and variable rim enhancement are visible on contrast-enhanced imaging.*

• *DWI-b800 may prove useful to predict early tumour recurrence.*

• *Post-IRE examinations reveal an initial volume increase followed by a decrease.*

**Electronic supplementary material:**

The online version of this article (doi:10.1007/s00330-016-4581-2) contains supplementary material, which is available to authorized users.

## Introduction

Patients with pancreatic cancer have a poor prognosis. For nonmetastatic disease, the only curative opportunity is surgical resection, and unfortunately, only 10–20 % of patients are surgical candidates [1]. Up to 40 % of patients present with nonmetastatic, but unresectable disease due to vascular encasement (locally advanced pancreatic carcinoma [LAPC] or American Joint Committee on Cancer [AJCC] stage III disease) [1, 2]. In recent years, image-guided pancreatic tumour ablation has gained increased interest when surgical options are excluded. Nevertheless, thermal ablation techniques are associated with substantial morbidity and mortality, due to the proximity of large vessels, the pancreatic and common bile duct, and the gastroduodenal wall [3]. Also, the so-called heat-sink effect can impede complete ablation [4].

Recently, irreversible electroporation (IRE) has emerged as a novel ablation technique that potentially circumvents the abovementioned limitations. IRE induces an electric field across cells to alter the cellular transmembrane potential. After reaching a sufficiently high voltage, the phospholipid bilayer structure of the cell membrane is permanently disrupted, inducing apoptosis. It is hypothesized that IRE leaves supporting tissue largely unaffected, preserving the structure of large blood vessels and bile ducts [5]. Since IRE relies on electrical energy, its efficacy is unaffected by the heat-sink effect. This suggests safer and more effective ablation of neoplasms adjacent to large vessels or fragile structures [6].

Multiple studies have suggested the safety and feasibility of pancreatic IRE [7–9], but only few have focused on ablation zone imaging characteristics and volumetry post-IRE in the clinical setting [10–12]. Familiarity with post-interventional imaging is essential to determine ablation success and for the detection of recurrence. Since isolated recurrence may be favourable over distant metastasis for patients’ prognosis, accurate imaging interpretation following IRE is of considerable importance [13].

The purpose of the present study was to assess specific imaging characteristics after percutaneous irreversible electroporation (IRE) for locally advanced pancreatic carcinoma (LAPC) with multiphasic contrast-enhanced (ce)MRI and ceCT. Additionally, imaging features prognostic for local recurrence were explored. The secondary aim was to quantify tumour and ablation zone volumes.

## Methods

Qualitative and quantitative analyses of imaging data were performed on all patients treated with percutaneous IRE for LAPC in the prospective PANFIRE-trial (Clinicaltrials.gov: NCT01799044). All patients gave written informed consent. The local institutional review board gave approval. Study design and conduct were in accordance with the guidelines for Good Clinical Practice.

### Patients and tumours

Between January 2014 and June 2015, 25 patients (12 men, 13 women; median age, 61 years [range, 41-78]) with histologically proven LAPC who met inclusion criteria were included. Prior to study enrolment, all participants were discussed in the multidisciplinary pancreatic tumour board. Inclusion criteria were radiologic confirmation of LAPC stage III (axial diameter ≤5 cm), American Society of Anaesthesiologists (ASA) performance status 1-3, and adequate bone marrow, liver, and renal function (Table 1A). Exclusion criteria were distant metastases, history of epilepsy or ventricular arrhythmias, an implanted stimulation device, and a metal biliary stent.

### IRE procedure

All procedures were performed by an interventional radiologist (MRM) under general anaesthesia as described previously [14]. A ceCT, using multiplanar image reconstruction, was made to define the three-dimensional tumour measurements. Size and shape, including a 5-mm margin, determined the number and configuration of the electrodes (NanoKnife, AngioDynamics, Latham, NY, USA). Three to six electrodes with an exposure length of 15 mm were placed in the outer border or just outside the tumour under CT-guidance. Ablation was performed between all electrode pairs that were separated between 15-24 mm from each other. For larger tumours, the needles were repositioned for one or more overlapping ablations (Table 2A). Per electrode pair a total of 100 pulses of 1500 V/cm and 90 μs were delivered. The AccuSync cardiac synchronization device (Accusync Research Monitor, Milford, CT, USA) was used to synchronize the electric pulses with the patient’s electrocardiogram.

### Imaging

CeMRI and ceCT scans were performed according to schedule (Table 1). MRI was performed using a 1.5-Tesla MRI (Signa HDxt, General Electric, Cleveland, OH, USA) with an 8-channel phased array coil. Imaging protocol included T2-weighted fast-recovery fast spin echo images (matrix 320⨯224; field of view [FOV] 400 mm; slice thickness 7 mm), diffusion-weighted images (DWI) (b0, b50, and b800 s/mm^2^; matrix 160⨯128; FOV 400 mm; slice thickness 8 mm) and breath-hold unenhanced and contrast-enhanced T1-weighted three-dimensional fat-suppressed spoiled gradient-echo images (matrix 256⨯256; FOV 350 mm; slice thickness 3 mm; respectively, matrix 256⨯224; FOV 400 mm; slice thickness 4.4 mm) in the arterial phase (20 s), portal venous phase (60 s), and delayed phase (120 and 180 s) after intravenous injection of gadolinium (Dotarem, Guerbet, Villepinte, France) in a dose of 0.2 mL/kg at 3 mL/s. CT data were acquired using a 64-row MDCT system (Siemens Sensation, Erlangen, Germany). Scanning parameters were 120 kV, 180 mAs, and 380 mm FOV. CT was performed after intravenous administration of a 100 mL bolus of non-ionic iodinated contrast material (Xenetix 300, Guerbet, Villepinte, France), at 4 mL/s with a scan delay of 40 s for the pancreatic phase and 70 s for the portal venous phase.

**Table 1:** Imaging schedule before and after IRE

0-2 weeks pre-IRE		ceMRI
IRE-procedure	ceCT	
+1 day		ceMRI
+2 weeks		ceMRI
+6 weeks	ceCT	ceMRI
+3 months*	ceCT	

*After 3 months of follow-up, ceCT was performed every 3 months

### Tumour and ablation zone evaluation

Two experienced abdominal radiologists (MCM and JEB) interpreted the ceCT and ceMR images independently. Per sequence, findings were graded systematically according to the specific tumour and ablation zone imaging characteristics compared to the surrounding healthy pancreatic parenchyma, using a region of interest (ROI). MRI intensity was evaluated on an ordinal 7-point scale (---/0/+++). CT density was assessed on an ordinal 3-point scale (-/0/+). Furthermore, the presence and configuration of periablational rim enhancement, intralesional gas pockets, and blood residues was evaluated. Discrepancies between the interpreters’ findings were solved by consensus.

Radiologic response was evaluated through Response Evaluation Criteria in Solid Tumours (RECIST) [15], in which recurrence was defined as a focal or diffuse growing mass within 1 cm of the ablated region compared to the new baseline-scan at 6 weeks post-IRE, accompanied by a substantial cancer antigen (CA) 19.9 rise (duplication compared to baseline). Tumours recurring within 6 months were considered early recurrences. Histopathologic confirmation was only obtained if patients were eligible for retreatment. To detect possible early imaging markers for treatment failure, a reassessment of the recurrence area was performed.

### Predicted and obtained treatment zone volumes

**Fig. 1:**
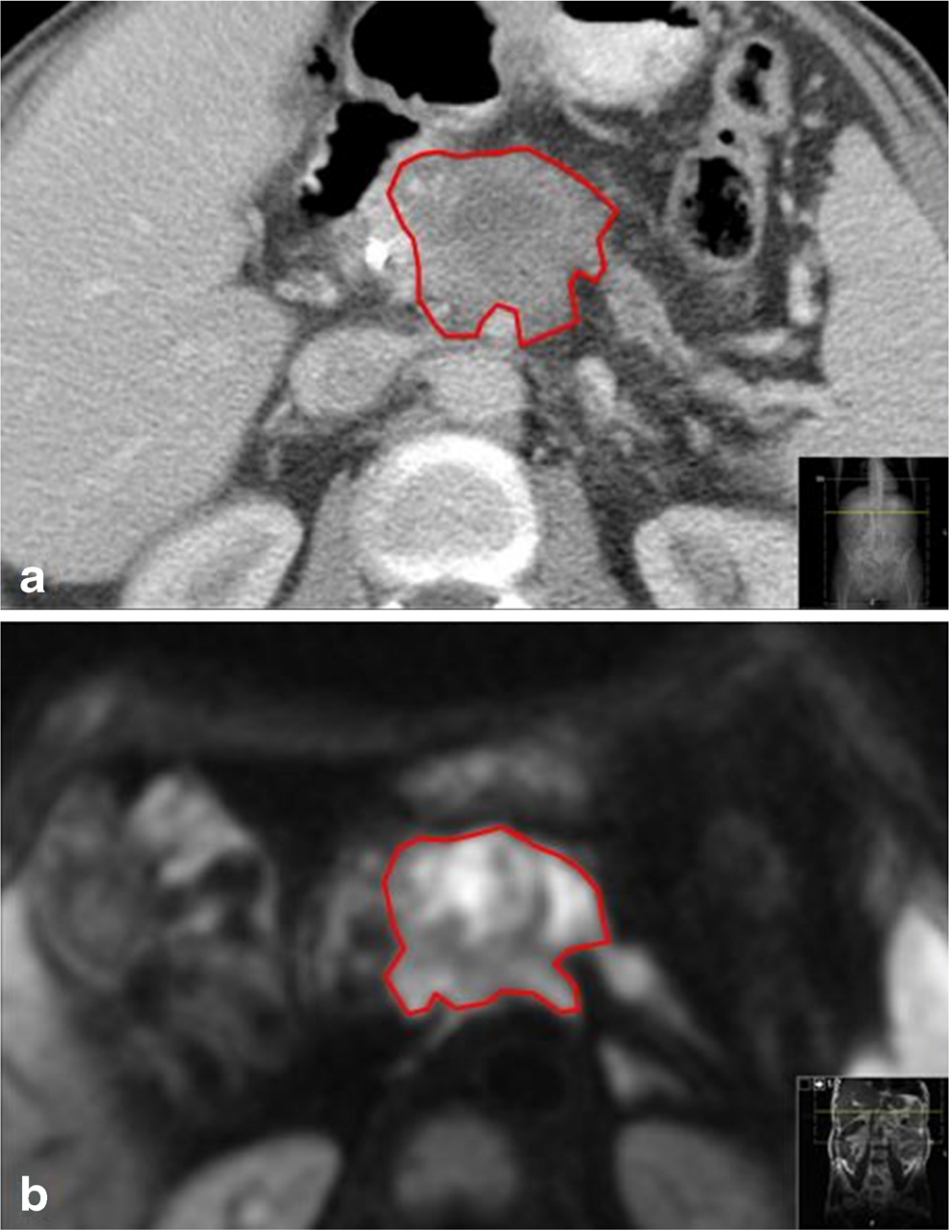
Manually drawn boundary of tumour on (**a**) ceCT (portal venous phase) and (**b**) DWI-b800 sequence

Tumour and ablation zone volumes were measured by manually drawing the boundary of the tumour and ablation zone on each portal venous ceCT and ceMRI DWI-b800 slice (Fig. 1). The volume of the segmented lesion resulted from the sum of all segmented slice surfaces, multiplied by the reconstruction increment (caliper method) [16]. Patients who developed an early recurrence were excluded from volumetric analysis.

### Statistical analyses

Descriptive statistics were used to present results as absolute numbers (normal distribution), median and range (non-normal distribution), or frequencies and percentages (categorical variables). The two-tailed Wilcoxon signed-rank test was performed to compare contrast ratio scores across sequences between pre- and post-treatment. Statistical analyses were performed using SPSS, version 20.0 (SPSS, Chicago, IL, USA). The level of statistical significance was set to *p* < 0.05. Interobserver-agreement was assessed with k-statistics [17]. The prognostic accuracy of a focal hyperintense spot on the 6-week follow-up DWI-b800 for the development of recurrence within 6 months post-IRE was determined with sensitivity, specificity, negative predictive value (NPV), and positive predictive value (PPV).

## Results

Twenty-five patients with a median age of 61 years (range 41-78) were included for analysis. Tumours were located in the pancreatic head (*n* = 18), body (*n* = 2), and uncinate process (*n* = 5). Needle placement and pulse delivery was successfully performed in all patients. Complications post-IRE were oedematous pancreatitis (*n* = 1), duodenal wall ulcer directly adjacent to the ablation zone (*n* = 1), new-onset biliary obstruction (*n* = 3), cholangitis with infected biloma (*n* = 1), and subtotal occlusion of the superior mesenteric artery (*n* = 1).

### CeMR imaging

Complete MRI follow-up was accomplished in 21 patients. Four patient were excluded from MR follow-up because of claustrophobia. CeMRI tumour and ablation zone findings are shown in Table 2. Interobserver agreement was substantial to excellent on DWI-b800, precontrast T1- and postcontrast T1-weighted MRI and moderate to excellent on T2- and postcontrast T1-weighted MRI (Table 3).

**Table 2:** Specific tumour and ablation zone imaging characteristics on each MRI sequence (median score). +, ++, +++ = hyperintense; 0 = isointense; -, --, --- = hypointense

	Pre-IRE	1 day post-IRE	2 weeks post-IRE	6 weeks post-IRE
T2	+	+	+	+
DWI-b800	++	+	+	+
ADC	-	0	0	0
T1 Precontrast	0	0	0	0
T1 Arterial phase	-	--	--	-
T1 Venous phase	-	---	---	--

**Table 3:**
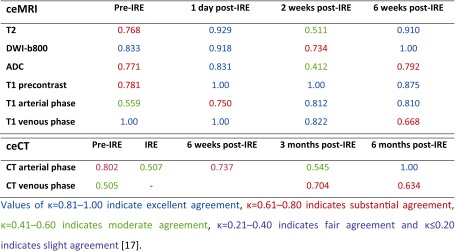
Interobserver agreement of ceMRI and ceCT sets (weighted k-values)

Prior to IRE, most tumours were markedly hyperintense on T2-weighted images (71 %, *n* = 15) and on DWI-b800 (86 %, *n* = 18) compared to surrounding normal pancreatic parenchyma. Pancreatic tumours appeared hypointense in 86 % (*n* = 18) on both apparent diffusion coefficient (ADC) and arterial phase T1-weighted MRI, and in 76 % (*n* = 16) on the portal venous phase T1-weighted MRI.

**Fig. 2:**
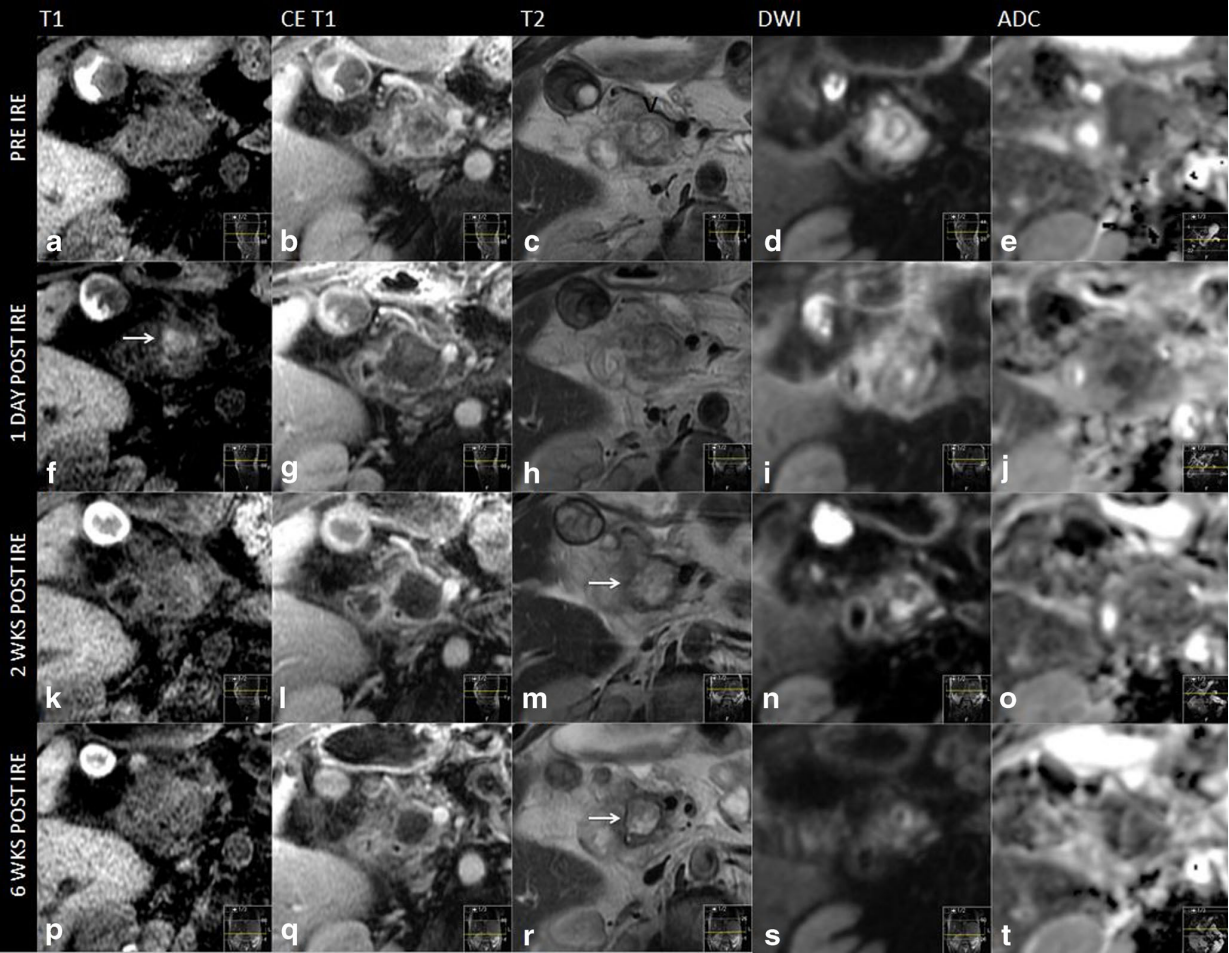
*Prior to IRE:* (**a**) Isointense tumour on T1 sequence (**b**) Hypointense (-) tumour on T1 sequence (portal venous phase) (**c**) Hyperintense tumour on T2 sequence (**d**) Hyperintense (++) tumour on DWI-b800 sequence (**e**) Hypointense (-) tumour on ADC map. *1 day post-IRE:* (**f**) Isointense IRE ablation zone with small hyperintense blood residues on T1 sequence (**g**) Hypointense (---) IRE ablation zone plus rim-enhancement surrounding the treated area on T1 sequence (portal venous phase) (**h**) Hyperintense (+) IRE ablation zone on T2 sequence (**i**) Hyperintense (+) IRE ablation zone on DWI-b800 sequence (**j**) Isointense IRE ablation zone on ADC map. *2 weeks post-IRE:* (**k**) Isointense IRE ablation zone on T1 sequence (**l**) Hypointense (---) IRE ablation zone plus rim-enhancement surrounding the treated area on T1 sequence (portal venous phase) (**m**) Hyperintense (+) IRE ablation zone plus hypointense rim enhancement surrounding the treated area on T2 sequence (**n**) Hyperintense (+) IRE ablation zone on DWI-b800 sequence (**o**) Isointense IRE ablation zone on ADC map. *6 weeks post-IRE:* (**p**) Isointense IRE ablation zone on T1 sequence (**q**) Hypointense (--) IRE ablation zone on T1 sequence (portal venous phase) (**r**) Hyperintense (+) IRE ablation zone plus hypointense rim enhancement surrounding the treated area on T2 sequence (**s**) Hyperintense (+) IRE ablation zone on DWI-b800 (**t**) Isointense IRE ablation zone on ADC map

Compared to original tumour intensity, one day post-IRE DWI-b800 MRI signal intensities notably decreased in all cases (*p* = 0.0002), accompanied by a subsequent ADC increase (*p* = 0.0044). At two and 6-week follow-up, intensity remained low on DWI-b800, in comparison with the initial lesion (*p* = 0.0022 and *p* = 0.0023, respectively) and high on ADC (*p* = 0.0010 and *p* = 0.0022, respectively). One day post-IRE, small areas of diffuse hyperintensity representing blood residues were detected in all ablated areas on precontrast T1-weighted images. At this point, the ablation zone contrast enhancement in the arterial and portal venous phase had decreased in all lesions as compared to initial tumour intensity (*p* = 0.0099 and *p* = <0.0001). In the portal venous phase, a hyperintense rim surrounding the IRE ablation zone was found in 71 % (*n* = 16) both 1 day and 2 weeks post-IRE, and was less often identified at 6-week follow-up (29 %, *n* = 6). At 2- and 6-week follow-up, tumour intensity remained low for the arterial phase (*p* = 0.0004 and *p* = 0.033) and portal venous phase (*p* = 0.0001 and *p* = 0.0009). On the T2-weighted sequences, ablation zone intensity during follow-up did not significantly differ from the initial tumour intensity. However, a remarkable hypointense rim surrounding the ablation zone was observed in 52 % (*n* = 11) of patients 2 weeks post-IRE on T2-weighted MRI. An example of typical MRI features is shown in Fig. 2, corresponding to successfully ablated tumours.

### CeCT imaging

**Fig. 3:**
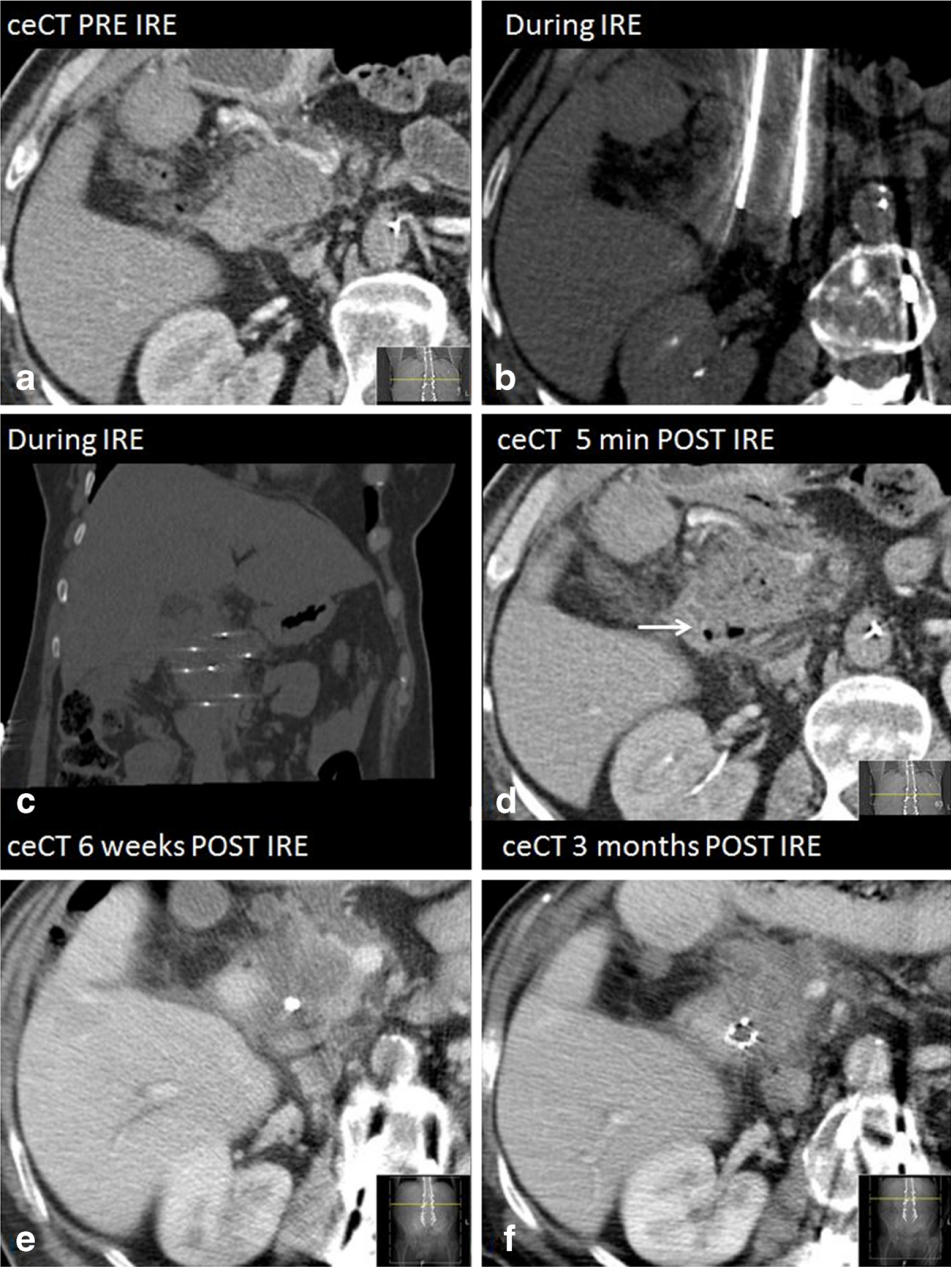
Imaging findings during follow-up on ceCT (**a**) Isoattenuating tumour on ceCT pre-IRE (**b**) CT-guided placement of electrodes around the outer border of the tumour (**c**) Confirmation of correct electrode configuration according to the treatment plan with a nonenhanced CT scan (**d**) Hypoattenuating IRE ablation zone with intralesional gas pockets immediately after IRE (**e**) Hypoattenuating IRE ablation zone at 6 weeks of follow-up (**f**) Hypoattenuating IRE ablation zone at 3 months of follow-up

Differences between attenuation pre- and post-IRE in the arterial and portal venous phase were not statistically significant. Table 4 and Fig. 3 show the tumour and ablation zone attenuation characteristics on ceCT. Interobserver agreement was mostly substantial to excellent (Table 3). Compared to the healthy pancreatic parenchyma the initial tumour appeared either isodense (56 %) or hypodense in the arterial phase (44 %) and hypodense (72 %) in the portal venous phase. Immediately after IRE, intralesional and periablational gas pockets were present in all cases. Post-IRE the ablation zones were primarily hypodense in the arterial phase after 6 weeks and 3 and 6 months (80 %, 52 %, and 56 %, respectively). In the portal venous phase 76 % of the ablated areas were slightly hypodense immediately post-IRE; at 6 weeks and 3- and 6-month follow-up, ablation zones were hypodense in 92 %, 92 %, and 94 %, respectively.

**Table 4:** Tumour and ablation zone signal densities on ceCT

	Pre-IRE		Post-IRE	+6 weeks post-IRE		+3 months post-IRE		+6 months post-IRE	
	Arterial	Venous	Venous	Arterial	Venous	Arterial	Venous	Arterial	Venous
Hypodense	11 *(44 %)*	18 *(72 %)*	19 *(76 %)*	20 *(80 %)*	23 *(92 %)*	14 *(52 %)*	23 *(92 %)*	9 (56 %)	15 (94 %)
Isodense	14 *(56 %)*	7 *(28 %)*	6 *(24 %)*	5 *(20 %)*	2 (8 %)	11 *(48 %)*	2 (8 %)	7 (44 %)	1 (6 %)
Gas pockets	-	-	25 (100 %)	-	-	-	-		

The data shown are number of patients (%). Pre-IRE = before IRE; post-IRE = immediately after IRE

### Early recurrence

**Fig. 4:**
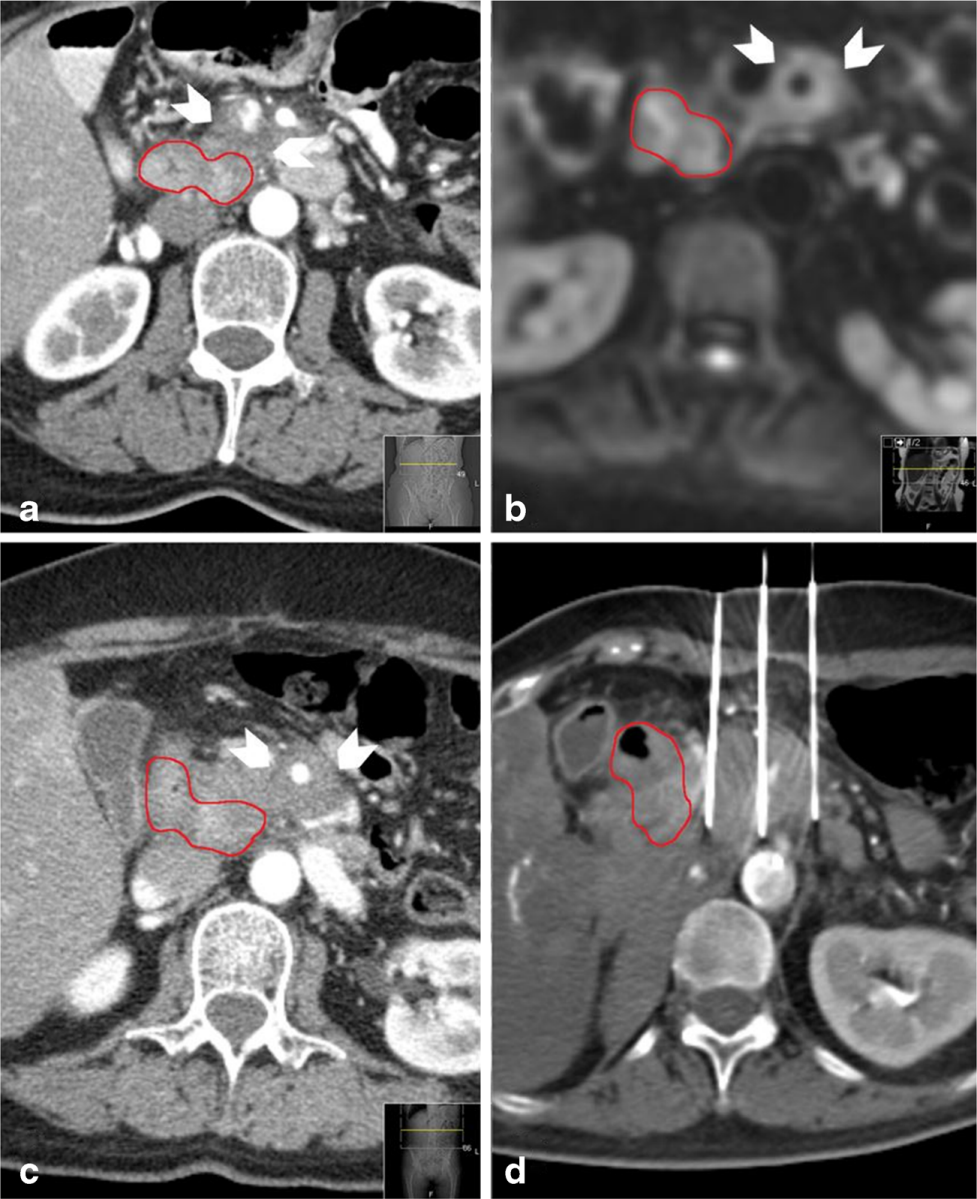
The development of a local recurrence. *Red line* = duodenum. **a** CeCT pre-IRE showing the initial tumour (*white arrowheads*) that was treated with IRE (**b**) MR DWI-b800 6 weeks post-IRE showing new hyperintensity around the superior mesenteric artery (*white arrowheads*) (**c**) CeCT 4 months post-IRE showing evident local recurrence (*white arrowhead*) (**d**) re-IRE of the local recurrence

**Fig. 5:**
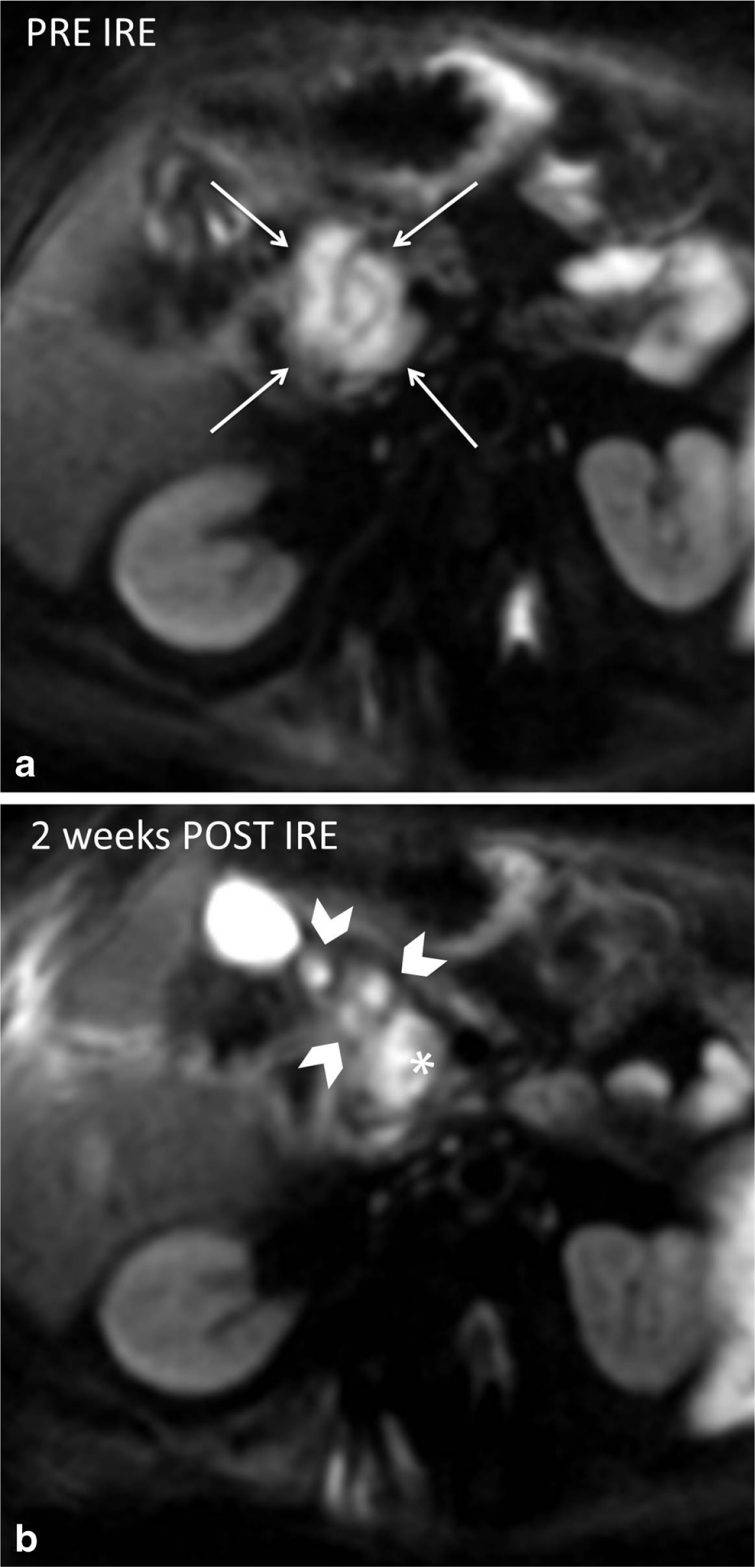
DWI b800 MR images showing (**a**) a hyperintensity of the tumour pre-IRE (*arrows*); (**b**) hypointensity of the ablated area with hyperintense marginal spots and a lymph node at 6 weeks post-IRE

During a median follow-up period of 6 months (range 3-17), five patients developed an early local recurrence at 2 (*n* = 1), 3 (*n* = 1), 5 (*n* = 2), and 6 (*n* = 1) months, detected on ceCT and accompanied by a substantial CA 19.9 rise. Histopathologic confirmation was obtained in one patient who was subsequently retreated with IRE (Fig. 4). The four remaining patients were considered unsuitable for retreatment because of excessive disease progression. Targeted analysis of the recurring areas revealed small hyperintense spots adjacent to the overall decreased ablation zone intensity on DWI-b800, which was low on ADC (Fig. 5). For this reason all DWI and ADC exams were prospectively re-assessed for the presence of these marginal spots by both reviewers (MCM and JEB). Interobserver agreement was substantial (k = 0.674). In 4/5 patients a marginal spot showing diffusion restriction correlated to early recurrence. In a fifth patient patchy hyperintensity at 6 weeks evolved into extensive local recurrence after 6 months. However, a marginal spot was also identified in 3/16 patients without early relapse (sensitivity 100 %, specificity 81 %, NPV 100 %, and PPV 63 %).

#### Tumour and ablation zone volumes

Ablation zones on both ceCT and DWI-b800 were difficult to delineate from the surrounding pancreatic parenchyma due to intralesional gas pockets, blood residues, and surrounding tissue oedema.

### CeMR imaging

The volume of one ablation zone prior to treatment and 6 weeks post-IRE could not be defined. Median tumour volume 0-2 weeks prior to intervention was 19 mL (range 6-58). One day post-IRE median ablation zone volume was 49 mL (range 16-100). At 2 weeks of follow-up, median volume was reduced to 16 mL (range 7-98). The median ablation zone volume remained mostly stable at 6 weeks of follow-up (14 mL, range 5-71) (Fig. 5).

### CeCT imaging

**Fig. 6:**
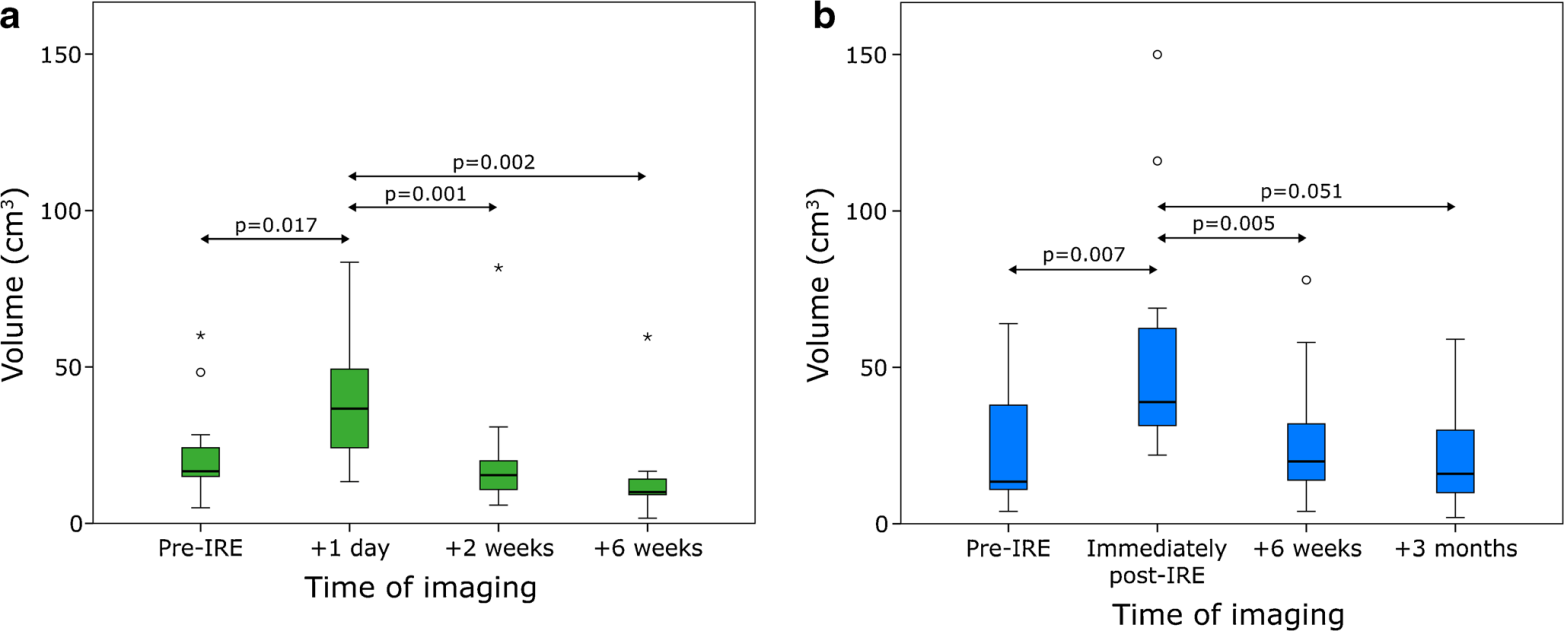
Box plots of tumour and ablation zone volumes pre- and post-IRE (**a**) MRI and (**b**) CT; *p*-values from Wilcoxon signed-rank test

Volumes prior to IRE (*n* = 2), immediately post-IRE (*n* = 8), 6 weeks post-IRE (*n* = 4), 3 months post-IRE (*n* = 1), and 6 months (*n* = 1) could not be precisely determined due to poorly demarcated margins of the ablation zone. A median tumour volume of 15 mL (range 4-98) was measured on ceCT pre-IRE. Median ablation zone volume directly after the intervention was 31 mL (range 19-150). On follow-up examinations after 6 weeks, median volume had decreased to 17 mL (range 2-59). Eventually, ablation zone volume was equal to original tumour volume at 3 months of follow-up (median 22 mL, range 4-78) and 6 months (median 28 ml, range 8-64) (Fig. 6).

## Discussion

Evaluation of tumour response after ablation is important to define treatment success and to guide future therapy [18]. Knowledge of postinterventional MR and CT findings is essential for accurate interpretation of the ablated area. Familiarity with these characteristics prevents confusion between normal or less typical postablational changes and residual or recurrent disease. In addition, timely recognition of IRE-related complications and vital tumour allows for expedited management and possible retreatment. In this study, the evolution of ablation zones based on ceMRI and ceCT was reviewed over a follow-up period of 90 days.

In the liver, compared to the surrounding normal parenchyma, a well-demarcated ablation zone is commonly visible on CT and MRI post-IRE [11, 19]. Also, IRE ablation zones in the liver depict a transient peripheral hyperenhancing rim [11, 19]. Since generally little healthy pancreatic tissue surrounds the pancreatic tumour, in our study the ablation zone was often ill-defined on MRI and especially on CT. Also, the presence of more oedema within the ablation zone often impeded precise ablation zone delineation.

Literature on post-IRE MRI is scarce and predominantly involves the liver; imaging data of pancreatic IRE in humans is not available yet. A reasonable explanation for the observed hyperintense rim surrounding the ablation zone post-IRE is reactive hyperaemia of oedematous inflammatory origin [20, 21]. However, it cannot be excluded that this rim still contains residual disease and longer follow-up is needed to explore the exact significance. The remarkable hypointense rim that we found on T2 at 2 weeks suggests hemosiderin deposition [22] resulting from degradation of the extravagated erythrocytes in the periphery of the ablation zone [23].

Post-IRE, arterial and portal venous phase CT attenuation decreased in nearly all patients. This decline in enhancement is in line with the observed postcontrast MRI findings, which may be indicative for accurate tumour therapy response [24]. The observed intralesional gas pockets may be caused by electrolysis of water into hydrogen and oxygen caused by the electric pulses [23], or by vaporization due to heat development, or by a combination of these mechanisms.

Initial post-IRE examinations revealed a notable volume increase on ceCT and ceMRI, followed by a decrease during follow-up. The calculated volumes varied widely between the two modalities, which is caused by the difficult ablation zone delineation from surrounding structures. Studies investigating the size and shape of the IRE ablation zone have predominantly correlated imaging findings to histology in animal studies. Overall, the radiological ablation zone size as measured on CT and MRI-DWI correlates well with the histological ablation zone [20, 25, 26]. In addition, studies suggested that ablation zone size and shape depend on the IRE parameters used and on the type of tissue ablated [27]. There is clear concordance between our findings and preclinical and early clinical studies that describe a reduction of the size of the ablated area over several weeks [6, 11, 12, 20], resulting from the clearance of cellular debris aided by the preservation of larger vessels [6, 20].

The World Health Organization (WHO) criteria and RECIST criteria, depend on decrease in tumour size [28, 29]. However, decrease in viable cell mass is not always reflected by changes in tumour size [30]. Exclusive reliance on tumour size does not, therefore, provide a complete assessment of tumour response and may lead to inaccurate conclusions [29]. A preferable method of post-IRE treatment evaluation is to combine tumour and ablation zone sizes with functional information such as alterations in enhancement and diffusion [31].

CT is the standard imaging modality used for follow-up of pancreatic cancer and has an accuracy of 93.5 % for detecting locally recurrent tumour after pancreaticoduodenectomy using RECIST [32]. Since all five patients showed DWI-b800 hyperintensity and low ADC values at the site of eventual recurrence at 6 weeks, DWI-b800 and ADC may be useful to predict early recurrence or incomplete ablation, similar to imaging after hepatic ablation [33]. This may allow for earlier retreatment. However, the presumed hemosiderin deposition mentioned above may limit the capability of DWI-b800 to interpret the ablated area, in particular when the treatment zone is small, as susceptibility effects may obscure small areas of recurrence or create false-positives. Clearly, larger numbers are needed to validate our finding. More on the oncologic outcome of this trial will be published separately in the near future.

^18^F-fluorodeoxyglucose positron emission tomography (^18^F-FDG PET) CT has demonstrated better diagnostic accuracy compared with ceCT [34] and even MRI (without DWI-b800) [35] in the diagnosis of pancreatic cancer. Also, ^18^F-FDG PET is increasingly used to assess tissue response to chemoradiation for LAPC. A recent study showed the difference in maximum standardized uptake value (SUV_max_) pre- and post-chemoradiation for LAPC was an independent predictor of clinical outcome.[34] In this study, ^18^F-FDG PET was not performed, but the value of ^18^F-FDG PET as a predictor for ablation success after pancreatic IRE should be investigated in future studies.

The greatest limitation of this study was the sample size, which precluded a meaningful quantitative data-analysis with respect to recurrences, therefore, no multivariable analyses were considered. Furthermore, histopathologic confirmation of recurrence was obtained in only one patient. Given the lack of clinical consequences and the associated risk of biopsy, no histopathologic confirmation was obtained in the remaining four patients. Another drawback was the often poorly delineated ablation zone caused by a peri-ablational inflammatory response, which renders the accuracy of the calculated volumes uncertain, especially on CT.

In conclusion, the most remarkable signal alterations after pancreatic IRE are shown by DWI-b800 and postcontrast T1-weighted MRI and these imaging characteristics may be useful to predict complete ablation and early recurrence. Future studies should elaborate whether imaging characteristics post-IRE can predict treatment outcome and stratify patients for potential retreatment. Currently we are performing a multicenter phase-III trial comparing IRE with stereotactic ablative body radiotherapy after neoadjuvant FOLFIRINOX (CROSSFIRE-study, registered at clinicaltrials.gov NCT02791503). Within this study all pre- and post-IRE images will be evaluated to validate present findings.

## Electronic supplementary material

Below is the link to the electronic supplementary material.

ESM 1 (DOCX 16 kb)

ESM 2 (DOCX 17 kb)

## Abbreviations

ADC: apparent diffusion coefficient
AJCC: American Joint Committee on Cancer
ASA: American Society of Anaesthesiologists
CA 19.9: Cancer antigen 19.9
ce: contrast-enhanced
CT: computed tomography
DWI: diffusion-weighted imaging
^18^F-FDG PET: ^18^F-fluorodeoxyglucose positron emission tomography
IRE: Irreversible electroporation
LAPC: Locally advanced pancreatic carcinoma
MRI: magnetic resonance imaging
NPV: negative predictive value
PPV: positive predictive value
RECIST: Response Evaluation Criteria in Solid Tumours
ROI: region of interest
SUV_max_: maximum standardized uptake value
WHO: World Health Organization
