# Supplementary material for: MR and CT imaging characteristics and ablation zone volumetry of locally advanced pancreatic cancer treated with irreversible electroporation
Source: Eur Radiol. 2016 Sep 22;27(6):2521–31. doi: 10.1007/s00330-016-4581-2 (PMC5409808; doi:10.1007/s00330-016-4581-2)
Supplement: Supplementary file 1 — (DOCX 16 kb) [file 330_2016_4581_MOESM1_ESM.docx]

**Table 1A.** Parameters of adequate bone marrow, liver and renal function

| **Hemoglobin** | ≥ 5.6 mmol/L |
| --- | --- |
| **Absolute neutrophil count (ANC)** | ≥ 1,500/mm^3^ |
| **Platelet count** | ≥ 100*10^9^/l |
| **Total bilirubin** | ≤ 1.5 times the ULN |
| **ALT and AST** | ≤ 2.5 x ULN |
| **Serum creatinin** | ≤ 1.5 x ULN  *or*  a calculated creatinine clearance ≥ 50 ml/min |
| **Prothrombin time or INR** | < 1.5 x ULN |
| **Activated partial thromboplastin time** | < 1.25 x ULN * |

ULN = upper limit of normal
* Therapeutic anticoagulation therapy is allowed if this treatment can be interrupted as judged by the treating physician.
