# Supplementary material for: MR and CT imaging characteristics and ablation zone volumetry of locally advanced pancreatic cancer treated with irreversible electroporation
Source: Eur Radiol. 2016 Sep 22;27(6):2521–31. doi: 10.1007/s00330-016-4581-2 (PMC5409808; doi:10.1007/s00330-016-4581-2)
Supplement: Supplementary file 2 — (DOCX 17 kb) [file 330_2016_4581_MOESM2_ESM.docx]

**Table 2A. Procedure specifications per patient**

| **Patient No.** | **Tumor size (cm) *** | | | **Number of electrodes** |
| --- | --- | --- | --- | --- |
|  | **Width** | **Depth** | **Length** |  |
| **1.** | 4,0 | 3,0 | 3,0 | 4 + pullback |
| **2.** | 3,8 | 2,5 | 4,0 | 8 |
| **3.** | 2,7 | 2,9 | 3,3 | 3 |
| **4.** | 2,5 | 2,5 | 5,0 | 6 + pullback |
| **5.** | 2,2 | 2,0 | 3,8 | 4 |
| **6.** | 5,0 | 4,2 | 5,0 | 8 +pullback |
| **7.** | 4,4 | 2,0 | 2,3 | 5 +pullback |
| **8.** | 4,0 | 3,6 | 3,6 | 7 +pullback (2x) |
| **9.** | 3,9 | 3,6 | 3,6 | 4 + pullback |
| **10.** | 3,8 | 2,4 | 4,3 | 5 |
| **11.** | 3,7 | 2,7 | 3,5 | 5 + pullback |
| **12.** | 3,6 | 2,9 | 3,0 | 5 + pullback |
| **13.** | 3,5 | 2,5 | 2,5 | 6 + pullback |
| **14.** | 2,9 | 2,9 | 3,7 | 7 + pullback (2x) |
| **15.** | 2,9 | 2,9 | 3,7 | 5 + pullback |
| **16.** | 2,5 | 3,5 | 2,5 | 5 + pullback (2x) |
| **17.** | 3,9 | 3,0 | 3,0 | 5 + pullback |
| **18.** | 4,3 | 3,2 | 4,1 | 7 + pullback |
| **19.** | 3,6 | 2,1 | 3,5 | 7 + pullback |
| **20.** | 3,7 | 2,7 | 3,7 | 7 + pullback |
| **21.** | 4,4 | 4,0 | 2,0 | 6 + pullback |
| **22.** | 3,8 | 3,1 | 3,8 | 7 + pullback |
| **23.** | 5,0 | 4,5 | 3,9 | 9 + pullback |
| **24.** | 4,5 | 3,9 | 5,0 | 8 + pullback (2x) |
| **25.** | 2,9 | 4,5 | 3,9 | 6 + pullback (2x) |

* Measured on ceCT
